# Landslide susceptibility mapping using an entropy index-based negative sample selection strategy: A case study of Luolong county

**DOI:** 10.1371/journal.pone.0322566

**Published:** 2025-05-09

**Authors:** Kong Yuzhong, Wu Hua, Xu Chong, Sun Jingjing, Zhu Kangcheng, Zhang Chenguang, Zhou Jianwei, Xu Tong, Su Taijin, Zhang Zelin, Kong Hui

**Affiliations:** 1 Tibet University, Lhasa, Tibet, China,; 2 Bomi Geological Hazards Field Scientific Observation and Research Station of the Ministry of Education, Bomê, Nyingchi, Tibet, China; 3 National Institute of Natural Hazards, Ministry of Emergency Management of China, Beijing, China; 4 Key Laboratory of Compound and Chained Natural Hazards Dynamics, Ministry of Emergency Management of China, Beijing, China; 5 Hunan Provincial Institute of Land and Space Survey and Monitoring, China; Hanoi University of Mining and Geology, VIET NAM

## Abstract

Landslides constitute a significant geological hazard in China, particularly in high-altitude regions like the Himalayas, where the challenging environmental conditions impede field surveys. This research utilizes the IOE model to refine non-landslide samples and integrates it with multiple machine learning models to conduct a comprehensive assessment of landslide susceptibility in Luolong County, Tibet. The IOE model objectively assigns weights to conditioning factors based on the degree of data dispersion, thereby enhancing the predictive accuracy when combined with machine learning models. This research employed Google Earth satellite imagery to construct a comprehensive database comprising 2517 landslide debris in Luolong County. Twelve conditioning factors were identified, encompassing geological environment, topography, meteorology, hydrology, vegetation, soil, and human activities. The IOE model was integrated with SVC, MLP, LDA, and LR models to systematically evaluate landslide susceptibility in Luolong County. The results demonstrate that, after optimizing the non-landslide samples, the coupled models significantly outperformed the unoptimized models in terms of AUC, accuracy, precision, and F1 score. The ranking of classification performance and effect among the four coupled models is IOE-MLP > IOE-SVC > IOE-LR > IOE-LDA. Notably, the AUC value of the IOE-MLP coupled model increased from 0.8172 to 0.9747. Moreover, in the extremely high susceptibility zones, the IOE-MLP model had the highest landslide frequency ratio among the four coupled models, demonstrating the optimal classification performance and the best classification effect. The study identifies land use, elevation, and slope as the predominant controlling factors conditioning landslides in Luolong County. The regions with the highest susceptibility to landslides in Luolong County are predominantly situated in the central areas near rivers and roads, whereas the areas with the lowest susceptibility are largely located in the southwestern, northern, and certain central regions at elevations above 4500 m, which are consistently shrouded in snow and ice. This comprehensive method effectively resolves the challenge of selecting non-landslide samples, thereby improving the predictive accuracy of the landslide susceptibility model. The results of this study offer significant insights for disaster prevention, mitigation, and land use planning in analogous geological settings.

## Introduction

As per the “14th Five-Year Plan for the Prevention and Control of Geological Disasters” issued by the Ministry of Land and Resources of China, geological disasters in our country are highly susceptible, frequent, and widespread. By the end of 2020, a total of 328,654 registered geological disaster hazard points were documented nationwide, posing a potential threat to the safety of 13.99 million people and 605.3 billion yuan in property[1]. Landslides are the most extensively distributed and numerous types of geological disaster in China, with the Himalayan region of Tibet being a focal area for the prevention and control of high-position remote chain landslides [2]. The Himalayan region is marked by steep mountains, high altitudes, thin air, and perennial snow and ice cover, leading to extremely adverse geological conditions. Many areas are inaccessible to human efforts, making it crucial to employ integrated remote sensing techniques to pre-evaluate landslide susceptibility and delineate different levels of targeted areas [3].

At present, the primary models employed in landslide susceptibility analysis are experience-driven models, physics-driven models, and data-driven models. Experience-driven models, which depend heavily on expert experience, are significantly influenced by subjective factors, thereby restricting their applicability. Physics-driven models necessitate comprehensive hydrological and geotechnical data, rendering them unsuitable for large-scale landslide susceptibility assessments. Owing to their objectivity and broad applicability, data-driven models have garnered favor among numerous scholars and experts.

Data-driven models can be further categorized into traditional statistical analyses and a variety of machine learning methods [4].Statistical models investigate the relationships between landslide debris and their conditioning factors, emphasizing the derivation and interpretability of the models, thereby more accurately capturing the spatial relationships of landslide samples. Commonly utilized statistical models include the Analytic Hierarchy Process (AHP) [5,6], Frequency Ratio Method [7], and the Index of Entropy (IOE) [8]. The AHP is highly susceptible to subjective biases, whereas the Frequency Ratio Method, while capable of objectively characterizing the relationships between conditioning factors and landslides, does not yield weight values for each conditioning factor. The IOE method objectively determines attribute weights based on the inherent dispersion of the data. It automatically normalizes the data during computation, obviating the need for preliminary standardization or normalization, thereby streamlining the calculation process and rendering it ideal for large datasets. Consequently, integrating the IOE model with other models can effectively minimize the impact of subjective biases and mitigate overfitting in machine learning. Machine learning models emphasize the exploration of relationships and structures inherent in the data, prioritizing predictive accuracy and optimization performance, thereby achieving superior predictive outcomes. Presently, models that have demonstrated notable success include Random Forests (RF) [9], Multilayer Perceptron (MLP) [10,11], Support Vector Classification (SVC) [12,13], Linear Discriminant Analysis (LDA) [14], and Logistic Regression (LR) [15]. Each of these models has its own strengths and weaknesses, yet all demonstrate strong generalization capabilities. However, individual models exhibit limited capacity to handle non-linear relationships, are unable to fully capture the complexity of landslide susceptibility and are prone to overfitting. Coupling statistical models with machine learning models can balance the spatial representation of landslides, more comprehensively capture data characteristics, reduce the risk of overfitting, and enhance prediction accuracy [16,17]. The Random Forest (RF) model is particularly sensitive to noisy data and may overfit the noise. Therefore, this study selects the Multi-Layer Perceptron (MLP) and Support Vector Classifier (SVC) models, which perform well with non-linear data, and the Linear Discriminant Analysis (LDA) and Logistic Regression (LR) models, which perform well with linear data, to be coupled with the IOE model to improve model accuracy and performance.

The expression of landslide susceptibility is influenced by the type of mapping unit, the algorithm type and its parameter optimization, and the choice of samples [18]. The more rational and accurate the mapping unit, the more effectively it can capture the terrain and geological conditions, thereby improving the precision of landslide susceptibility evaluations. The two predominant mapping units in use are grid cells and slope units. While slope units can partially represent terrain and geological environments, they may manifest as jagged or linear shapes, necessitating adjustments based on actual topography and geomorphology. This adjustment process is labor-intensive and susceptible to errors. Although the grid cell method does not capture terrain and environmental nuances, it provides extremely straightforward and efficient calculations, enabling the delineation of extensive regions, which is currently the most prevalent method[19]. When the grid cell size is reduced to less than 2000m, machine learning techniques applied to landslide susceptibility assessments exhibit enhanced predictive accuracy [20]. Various algorithm types of process data differently and have distinct applications. Logistic regression, linear regression, and linear discriminant analysis are well-suited for linear relationships, while support vector machines, decision trees, and neural networks are adept at handling nonlinear relationships. Algorithmic parameter optimization enhances model performance, reduces computational resource consumption, and improves model interpretability. Utilizing Bayesian algorithms for parameter optimization of logistic regression and random forest models can markedly improve the accuracy of landslide susceptibility predictions. The optimization of the AdaBoost model through recursive feature elimination and particle swarm optimization can significantly enhance the predictive effectiveness of landslide susceptibility [21]. The application of Bayesian optimization techniques for hyperparameter tuning of logistic regression and random forest models has resulted in substantial improvements in their predictive performance for landslide susceptibility [22].

While algorithms and mapping units are significant, in machine learning, the selection and quality of samples are paramount. This is because, regardless of the algorithm’s sophistication, issues with the sample data (such as noise, bias, imbalance, etc.) can adversely impact the model’s performance and predictive accuracy. Furthermore, the quality of samples directly influences the model’s learning effectiveness and generalization capabilities, whereas algorithms and mapping units primarily dictate how information is extracted from the samples and how the model is constructed. Consequently, landslide samples should fully encapsulate the diverse conditions and characteristics associated with landslide events. These samples can be derived from remote sensing image analysis or field investigations, thereby ensuring a high degree of accuracy. Non-landslide samples should be carefully selected to minimize the inclusion of potential landslide sites and are generally obtained indirectly through various methods, as direct acquisition is not feasible. Researchers have yet to establish a standardized approach for selecting non-landslide samples. Presently, three primary methods are employed [23]. The first is random sampling, which is extensively utilized. Some researchers directly choose non-landslide points outside the landslide areas, which may share similar geological characteristics with the landslide points [24]. Furthermore, other scholars create buffer zones around landslide points and select non-landslide points beyond these zones. While random sampling is straightforward, it can lead to an uneven distribution of non-landslide samples, thereby compromising the model’s accuracy. The determination of buffer distances is highly subjective, and the non-landslide points selected may lack precision[25,26]. The second category involves selecting non-landslide samples based on factor constraints, which involves choosing non-landslide samples outside the range of environmental factors associated with landslide occurrences. For example, non-landslide points are selected in low-slope areas[27,28], using the target space outward sampling method[29], and selecting non-landslide points through the optimization of environmental factors[30]. When employing this method, selecting only one environmental factor may overemphasize its role, resulting in unreliable outcomes. Conversely, selecting multiple or all environmental factors can introduce noise, which also compromises the accuracy of the results. The third category involves selecting non-landslide samples through coupled models, which represents one of the more sophisticated approaches to negative sample selection. Utilizing random selection and information value methods to identify non-landslide samples, it has been demonstrated that the information value method can markedly enhance sample quality and model accuracy [31]. When employing random selection, buffer zone, frequency ratio, and analytic hierarchy methods to select non-landslide samples, it has been noted that the analytic hierarchy method yields the best results, followed by the frequency ratio method, with the random selection method being the least effective [32]. Integrating the certainty coefficient method with multi-kernel support vector machines for landslide susceptibility evaluation can produce outstanding results [33]. In conclusion, the adoption of a coupled model to identify non-landslide samples yields higher-quality and more diverse samples, consequently leading to a notable increase in the model’s accuracy[34].

This study initially utilizes satellite imagery from the Google Earth platform, employing a human-computer interactive visual interpretation technique to analyze landslide debris in Luolong County and create a comprehensive landslide relic database. It then integrates the advantages of the IOE model with those of machine learning models, applying IOE-LDA, IOE-MLP, IOE-SVC, and IOE-LR coupled models to perform a detailed comparative analysis of the landslide debris in Luolong County. Subsequently, it employs the area under the Receiver Operating Characteristic Curve (AUC), accuracy (Acc), precision, F1 score, and landslide frequency ratio to assess the overall effectiveness of each model, thereby developing a more appropriate method for selecting non-landslide samples and evaluating landslide susceptibility in high-altitude cold regions.

## Materials and methods

### Study area

**Fig 1:**
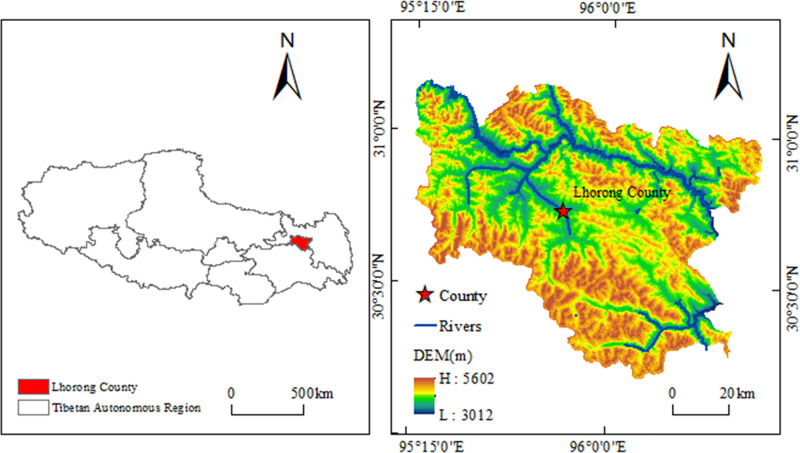
Location of the study area.

Luolong County is situated in the northeastern part of the Tibet Autonomous Region and the southwestern part of Changdu City, at the southeastern extremity of the Nyenchen Tanglha Mountains and the upper reaches of the Nu River (Fig 1). It is positioned between 30°10’ N and 30°50’ N latitude, and 95°10’ E and 95°50’ E longitude, encompassing a total area of 8098.4 km² and an average elevation of approximately 3859 m [35].

Luolong County predominantly features high mountain and canyon topography, with lower elevations in the central area and significant variations in terrain. The region is interlaced with numerous rivers and abundant lakes, including the Nu River, the second-largest river in Tibet, which traverses the central and northern parts of the county. The southern part of the county is dominated by the Nyenchen Tanglha Mountains, which extend in a northwest-southeast direction, spanning over 100 km and are perpetually covered with snow and ice. Luolong County lies within a plateau temperate semi-arid monsoon climate zone, characterized by extended daylight hours, low temperatures, substantial diurnal temperature variations, minimal annual temperature differences, distinct dry and rainy seasons, and a

lengthy, cold winter. Annual rainfall ranges from 372 to 559 mm, predominantly occurring from June to September, with the maximum daily rainfall varying between 14.0 and 39.2 mm. Luolong County is situated at the convergence of the Gondwana and Cathaysian ancient continents, within the Cathaysian Tethyan tectonic domain, specifically in the Gangdise-Nyainqentanglha mountain sheet. The county hosts several fault zones, such as the Shuoban Duo Fault Zone, the Chada-Baqu Fault Zone, the Xinben Fault Zone, and the Baqu Dongcun Fault Zone. The exposed geological formations include the Carboniferous, Paleogene, and Quaternary systems. The exposed bedrock comprises the Paleogene Zongbai Group, the Upper Carboniferous and Lower Permian Laigu Group, and the Lower Cretaceous granite, with predominant lithologies of siltstone, black slate, gray metamorphic sandstone, conglomerate-bearing slate, and a minor amount of mafic volcanic rock [36].

Luolong County is typified by a classic “V” shaped deeply incised canyon landscape, with elevations descending from 5476 m to 3153 m, resulting in a vertical drop exceeding 2300 m. This steep gradient creates conditions that are highly conducive to landslides. The area is situated in the globally recognized seismic active zone (The Mediterranean-Himalayan seismic belt) and is traversed by multiple fault zones, causing rock fragmentation and facilitating the formation of landslides. Furthermore, Luolong County serves as a vital corridor for China’s second railway to Tibet (the Sichuan-Tibet Railway) and a key transportation route in southeastern Tibet. It is also a significant region for hydropower development, hosting projects such as the Xinrong, Maquyong, Zongzha Cuo, and Zhongyi hydropower stations. The development of these human-engineered projects may elevate the risk of landslides. In recent years, influenced by global climate warming, the freeze-thaw processes in the region have become more pronounced, thereby augmenting the potential for landslides. Consequently, undertaking a comprehensive investigation of landslides in Luolong County is crucial for safeguarding the life and property of local inhabitants and fostering regional economic growth.

### Data sources

#### Source of landslide relic database.

Landslide relic data is of paramount importance for the study and mitigation of landslide disasters. These data not only reveal the spatial distribution characteristics of landslides but also provide essential support for predicting the likelihood of landslides and assessing their risks. In landslide disaster research, the precision and comprehensiveness of data are critical. Therefore, effectively collecting and analyzing landslide relic data has become a key issue in research. Presently, there are two predominant methods for obtaining landslide relic data: automated landslide extraction and human-computer interactive visual interpretation [37]. Automated landslide extraction employs machine learning algorithms to discern the features of sample data, enabling automatic extraction [38]. However, its precision is contingent upon the type of remote sensing imagery, regional topographic characteristics, and the nature of the landslide, rendering it more appropriate for post-earthquake emergency activities. Visual interpretation harnesses human and temporal resources to precisely identify targets in complex topographic regions, yielding a comprehensive and accurate database [39,40]. Through visual interpretation, researchers can meticulously analyze each topographic feature, ensuring that every detail within the landslide area is accurately documented and categorized. While visual interpretation achieves relatively high accuracy in landslide identification, it is constrained by the interpreter’s professional knowledge and experience, introducing considerable subjectivity. Additionally, it is characterized by low automation, inefficiency, and high costs. The landslide identification method leveraging human-computer interactive interpretation technology can markedly improve the efficiency of automatic landslide identification compared to traditional visual interpretation. It minimizes human intervention, rendering landslide identification more intelligent and efficient [41,42].

**Fig 2:**
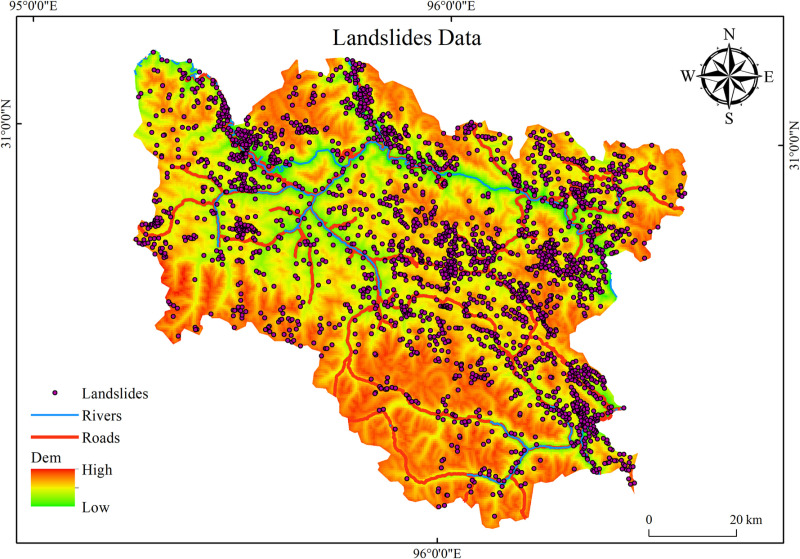
Landslide Relic Dataset.

This study employs remote sensing imagery from the Google Earth platform, segmenting the research area into smaller grid cells and utilizing a human-computer interactive visual interpretation technique for frame-by-frame analysis. This approach resulted in the creation of a landslide relic dataset containing 2517 landslide points, as depicted in Fig 2. The interpretation of landslides is primarily based

on the differences in spectral characteristics, shape, and texture relative to surrounding features, following this general procedure: Initially, objects with higher brightness are identified based on hue. Subsequently, regular artificial objects among the high-bright features are filtered out based on morphological characteristics. Next, interference from features such as agricultural land is eliminated using texture information. Finally employing manual methods to accurately determine the correctness of the identified landslides. During manual interpretation, the approximate location of the landslide is ascertained based on its morphological features and topographical context. Subsequently, the landslide boundary is delineated by contrasting the landslide with its surrounding environment. Ultimately, the precise location of the landslide center is established using the scarp and the landslide deposit [23,43]. Overall, the landslide debris in Luolong County are predominantly distributed along a northwest-southeast orientation, with a higher incidence of landslides in areas where rivers and roads are more concentrated, and relatively fewer landslides in regions with significantly higher surrounding elevations.

#### Data sources for landslide conditioning factors.

**Fig 3:**
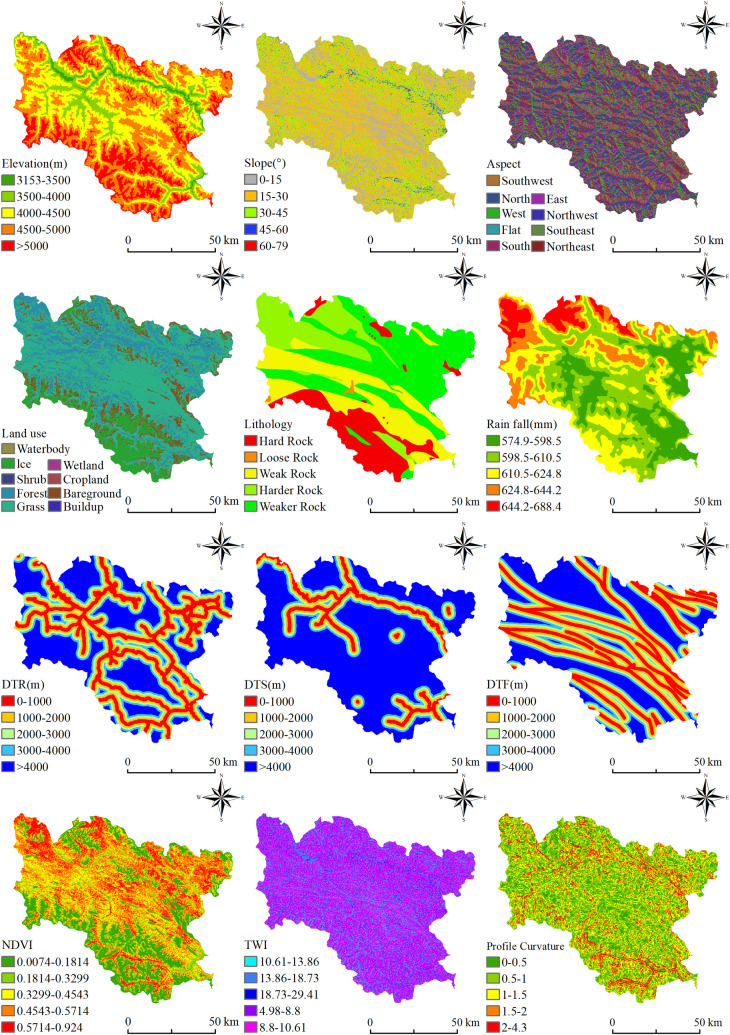
Grading diagram of conditioning factors.

Building upon the foundational work of previous researchers, this study identifies 12 conditioning factors that are closely associated with landslides across five key dimensions: geological environment, topography, meteorology, hydrology, vegetation and soil, and human engineering activities [44] (Fig 3). It establishes a comprehensive landslide susceptibility evaluation index system for Luolong County, with the data sources for each conditioning factor detailed as follows (Table 1).

**Table 1.** Data sources of landslide points and impact factors.

conditioning factor	Name of the data	Data type	Data source
Distance to faults	1:250,000 geological map of China	Raster	https://www.resdc.cn
Lithology distribution	Database of engineering geological rock groups on the Tibetan Plateau	Vector	https://data.tpdc.ac.cn
Elevation、Slope、Aspect、Profile curvature、TWI	30 m spatial resolution DEM data for China	Raster	http://www.gscloud.cn
Distance to roads and Distance to rivers	Basic geographic data of river systems, roads, and administrative boundaries	Vector	http://www.gscloud.cn
Rainfall	Multi-year average rainfall data for China	Raster	http://www.gisrs.cn
NDVI	30 m spatial resolution NDVI data for Luolong County in 2020	Raster	http://www.nesdc.org.cn
Land use	Land cover data of the Tibetan Plateau with a spatial resolution of 30 m (2000–2020)	Raster	https://data.tpdc.ac.cn

#### Conditioning factors.

In this study, all conditioning factors are uniformly projected using the Mercator projection with a 3-degree zone method for the 47N zone. Given that the resolution of the DEM, rainfall, NDVI, and land use data employed in this study is 30 meters, it is most appropriate to process the mapping units into 30m × 30m grids.

Elevation: Variations in elevation denote the terrain’s ridges or valleys, and changes in height result in alterations in slope. Rainfall and land use are also closely linked to elevation. The elevation data for Luolong County was derived from the national DEM digital elevation model with a spatial resolution of 30 m. The elevation spans from 3153 to 5476 m and is categorized into five levels: 3153–3500 m, 3500–4000 m, 4000–4500 m, 4500–5000 m, and > 5000 m, with areas above 4000 m comprising 85%.

Slope: Characterizes the inclination of the surface, indicating the steepness of the slope. A higher slope indicates a steeper surface, greater shear force in the rock mass, reduced stability of the slope body, and increased likelihood of landslides. The slope data are derived from the DEM with a spatial resolution of 30 m and are categorized into five levels: 0°-15°, 15°-30°, 30°-45°, 45°-60°, and > 60°, with the 15°-30° range comprising 49%.

Aspect: Broadly categorized into sunny and shady slopes, where slopes facing the sun are designated as sunny slopes, and those facing away from the sun are designated as shady slopes. Aspect indicates the accumulation and distribution of moisture on sunny and shady slopes, with sunny slopes typically being more susceptible to landslides. Aspect data are derived from the DEM with a spatial resolution of 30 m and are divided into nine categories: flat, north, northeast, east, southeast, south, southwest, west, and northwest.

Profile curvature: Profile curvature denotes the degree of curvature of the slope surface in the direction perpendicular to the slope, influencing the stress distribution and water accumulation on the slope, and thus indirectly affecting the risk of landslides. Profile curvature data are derived from the DEM with a spatial resolution of 30 m and are categorized into five levels: 0–0.5, 0.5–1, 1–1.5, 1.5–2, and 2–4.3, with the range of 0–1.5 comprising 82%.

Geological lithology: Geological lithology is a key internal factor in landslide development. The mechanical properties of different rock groups differ markedly, resulting in varying degrees of slope stability. Geological lithology is derived from the 1:500,000 geological map of the Tibetan Plateau, categorizing rock types into five groups: hard rock groups, moderately hard rock groups, moderately weak rock groups, weak rock groups, and loose rock groups. The moderately weak rock group covers the largest area, comprising 39%.

Distance to Fault (DTF): The fault structures within Luolong County predominantly develop in a north-south orientation, exerting a significant impact on the development of joints and fractures in geological bodies. The presence of faults results in rock fragmentation, destabilizing their structure and facilitating landslide formation. The degree of rock fragmentation is directly proportional to the distance from faults and fractures; the closer the distance to faults and fractures, the less stable the rocks become, and the greater the likelihood of landslides. Fault information is derived from geological maps, and buffer zones are established at 1000-meter intervals, categorized into five levels.

Rainfall: While the primary controlling factors for the formation of landslide geological hazards are the geological environment and topography, the intensity of rainfall is also a significant triggering factor for landslides. Rainfall increases the self-weight of the soil and the saturation of soil moisture, reducing the shear strength of the rock and soil mass, thereby elevating the risk of landslides. Moreover, rainfall can lead to increased surface runoff and rising groundwater levels, impacting the stability of the rock and soil mass. This study utilizes national annual average rainfall data from 1991 to 2020, categorized into five levels using the natural breaks method: 575–599mm, 599–611mm, 611–625mm, 625–644mm, and 644–688mm, with the 575–625mm range comprising 79%.

Distance to River (DTS): The proximity to rivers is a significant factor influencing landslides. Firstly, slopes adjacent to rivers are more prone to erosion and water level fluctuations, which can compromise slope stability. River erosion may diminish slope stability, and fluctuations in water levels can cause soil saturation, thereby elevating the risk of landslides. Secondly, regions near rivers are generally moist, facilitating the penetration of water into the slope, resulting in soil saturation and decreased stability, thus increasing the potential for landslides. This study derives river vector data from fundamental geographic data, creates buffer zones at 1000-meter intervals, and categorizes them into five levels.

Normalized Difference Vegetation Index (NDVI): Indicates the growth status of vegetation in a region. Lower NDVI values suggest sparser vegetation, characterized by shallow and sparse plant roots, which makes it challenging for vegetation to anchor the local soil, thus increasing the potential for landslides. This study employs national NDVI data with a 30-meter spatial resolution from 2020, spanning from -9999–9999. Dividing this data by 10000 results in NDVI values ranging from -1–1. The data is subsequently categorized into five levels using the natural breaks method: 0–0.18, 0.18–0.33, 0.33–0.45, 0.45–0.57, and 0.57–0.92, with the 0.33–0.57 range comprising 50%.

Topographic Wetness Index (TWI): TWI serves as a physical indicator of the influence of regional terrain on runoff direction and accumulation. A higher TWI value signifies a greater water content or potential soil moisture in the area. Increased soil moisture not only augments the self-weight of the slope but also diminishes the soil’s cohesion and angle of internal friction, thereby reducing the soil’s shear strength, enhancing the slope’s instability, and consequently elevating the risk of landslides. TWI is derived from the DEM with a spatial resolution of 30 m and is categorized into five levels using the natural breaks method: 5.10–8.70, 8.70–10.59, 10.59–13.75, 13.75–18.16, and 18.16–28.17, with the range of 5.1–10.59 comprising 81%.

Land use: Forests with high vegetation cover exhibit superior soil shear strength, thereby stabilizing slopes. In contrast, slopes resulting from excavated roads and mines, due to their low vegetation cover, have reduced soil shear strength, increasing the likelihood of landslides. This study employs land cover product data from the Qinghai-Tibet Plateau with a 30-meter spatial resolution from 2020, categorizing land use into nine classes based on original land categories: forests, shrublands, grasslands, wetlands, farmlands, built-up areas, glaciers, bare land, and water bodies, with grasslands comprising 57%.

Distance to Road (DTR): Slopes adjacent to roads are more susceptible to influences from road construction, drainage systems, increased loads, and traffic activities, potentially elevating the risk of landslides. This study derives road vector data from fundamental geographic data, creates buffer zones at 1000-meter intervals, and categorizes them into five levels.

### Data correlation analysis method

The landslide process is a gradual change process, influenced by a multitude of factors. Conditioning factors may sometimes display collinearity and strong correlation, resulting in data redundancy that impacts the accuracy of the evaluation model. Therefore, it is crucial to choose an appropriate method to detect correlations among factors and minimize data redundancy. Collinearity diagnostics are employed to identify severe collinearity issues among multiple independent variables in regression analysis. Typically, a Variance Inflation Factor (VIF) exceeding 5 and a tolerance below 0.2 signify the presence of multicollinearity [45]. Pearson correlation analysis is mainly utilized to evaluate the correlation between two variables. The greater the absolute value of the correlation coefficient (r), the stronger the linear relationship between the variables. Generally, an absolute value of r greater than 0.8 indicates a strong correlation between factors [46].

### Landslide susceptibility assessment method

#### (1) IOE model.

The IOE model [8] is a statistical approach that objectively quantifies the significance of each conditioning factor in relation to the indicator, determining the differences among the conditioning factors and their contributions to the evaluation index. The calculation steps and formulas are as follows:


$$\text{F}{\text{R}}_{ij}=\frac{\text{a}}{\text{b}}.$$ (1)

$$P_{ij}=\frac{FR_{ij}}{\sum_{j=1}^{\text{s}}FR_{ij}}$$ (2)

$$H_{i}=-\sum_{j=1}^{s}P_{ij}\times \log_{2}P_{ij}$$ (3)

$$H_{i\max}=\log_{2}S$$ (4)

$$I_{i}=\frac{H_{i\max}-H_{i}}{H_{i\max}}$$ (5)

$$P_{i}=\frac{1}{S}\sum_{j=1}^{s}FR_{ij}$$ (6)

$$W_{i}={\text{I}}_{i}\times {\text{P}}_{i}$$ (7)

In the formula, FR_ij_ denotes the frequency ratio of landslides for each conditioning factor across various interval ranges; a and b respectively indicate the ratio of landslide points and the area ratio for the conditioning factor within these intervals; P_ij_ signifies the probability density; S represents the count of intervals for each conditioning factor; H_i_ and H_i,max_ respectively signify the entropy value and the maximum entropy value; I_i_ denotes the information rate of each conditioning factor; W_i_ denotes the comprehensive weight of each conditioning factor.


$$LSI=\sum_{i=1}^{\text{n}}FR\times W_{i}$$ (8)

In the equation, LSI denotes the landslide susceptibility index.

#### (2) MLP model.

The MLP model [47] is a type of multilayer feed forward network within artificial neural networks, capable of highly parallel processing, exhibiting excellent fault tolerance and robust adaptive and self-learning capabilities. It primarily comprises an input layer made up of a set of perceptron units, one or more hidden layers composed of computational nodes, and an output layer consisting of computational nodes. Each layer of nodes is fully interconnected with the subsequent layer, such that any neuron in the preceding layer is connected to all neurons in the following layer. Each hidden layer node incorporates an activation function, and through the application of multiple layers of activation functions, it can transform linear rules, thereby enabling the recognition of nonlinear data. Consequently, it can address many nonlinear classification problems.

#### (3) SVC model.

The SVC model [48] is a robust machine learning algorithm designed for classification tasks. Its fundamental concept involves identifying an optimal hyperplane that segregates samples from different classes. This hyperplane not only aims to maximize classification accuracy but also seeks to maximize the margin between classes, which is the distance from the support vectors to the hyperplane. By locating this optimal hyperplane within the feature space, SVC enhances its generalization capability for new data. It can address nonlinear classification problems by employing kernel functions to project data into a higher-dimensional space.

#### (4) LDA model.

The LDA model [49] is a supervised learning technique utilized in statistics and machine learning, primarily for addressing classification and dimensionality reduction tasks. It utilizes the Fisher linear discriminant criterion to maximize the between-class scatter and minimize the within-class scatter, thereby identifying the optimal projection direction. This method ensures that data from different classes are maximally separated, while data from the same class are closely clustered, thereby facilitating classification.

#### (5) LR model.

The LR model [50] is a predictive technique employed in statistics and machine learning to tackle classification problems. Its principal entails utilizing a logistic function to convert the output of linear regression into values ranging from 0 to 1, thereby enabling binary classification. It is primarily composed of three steps:

(a) Employ a linear regression equation to estimate the value Z.

$$Z=\beta_{0}+\beta_{1}X_{1}+\beta_{2}X_{2}+\cdots +\beta_{n}X_{n}$$ (9)

In the formula, β is the coefficient, and X represents the feature.

(b) The Z value derived from the linear equation is converted into a value P ranging from 0 to 1 using the logistic function (Sigmoid function).

$$P=\frac{1}{1+e^{-z}}$$ (10)

(c) Classify according to the value of P. If P exceeds 0.5, predict it as the positive class; otherwise, predict it as the negative class.

#### (6) Coupled model.

The objective IOE model is distinguished by its high stability and consistency, allowing it to holistically account for the interrelations and impacts among various indicators. The IOE models integrated with SVC, MLP, LDA, and LA, through their optimization of non-landslide samples, decrease the likelihood of landslide samples being misclassified as non-landslide samples, thus improving the precision of the predictive models. The process is divided into two stages: The initial stage employs the landslide relic dataset as landslide samples, creates a no-landslide area by buffering the dataset by 1km, and subsequently selects an equivalent number of non-landslide samples from this area as non-landslide samples. The landslide and non-landslide samples are merged into sample set 1, and are analyzed using the SVC, MLP, LDA, and LA models individually, which are considered single models. In the second part, the landslide scar dataset was used as the landslide samples. An IOE model was employed to generate landslide susceptibility maps with five distinct susceptibility levels. The extremely low and low susceptibility zones were selected from these maps as non-landslide areas. Non-landslide samples, equal in number to the landslide samples, were randomly selected from these non-landslide areas with a minimum spacing of 500 meters to serve as non-landslide samples. The positive and non-landslide samples were then combined to form Sample Set 2, which was analyzed using the SVC, MLP, LDA, and LA models, thereby constituting the coupled models.

### Validation metrics

The ROC curve is frequently employed to assess the performance of classification models. The horizontal and vertical axes of the ROC curve represent specificity and sensitivity, respectively, indicating the proportions of correctly predicted non-landslide and landslide samples. The closer the ROC curve is to the upper left corner, the greater the area under the curve (AUC), signifying superior classification performance of the model, thus rendering the model more effective and precise. Furthermore, the confusion matrix in the field of machine learning is commonly utilized to compare classification outcomes with actual measured values. Each column corresponds to the predicted class, while each row corresponds to the true class. Acc, Precision, and F1 can be derived from the confusion matrix, where Acc denotes the proportion of correctly identified samples relative to the total samples, Precision denotes the proportion of truly landslide samples among those classified as positive by the model, and F1 signifies the harmonic meaning of precision. The formulas are as follows [51]:


$$Accuracy=\frac{\left(TP+TN\right)}{\left(TP+TN+FP+FN\right)}$$ (11)

$$\mathrm{Pr}ecision=\frac{TP}{\left(TP+FP\right)}$$ (12)

$$F1=\frac{2TP}{\left(2TP+FP+FN\right)}$$ (13)

### Landslide susceptibility assessment workflow

#### (1) Methodological Approach.

**Fig 4:**
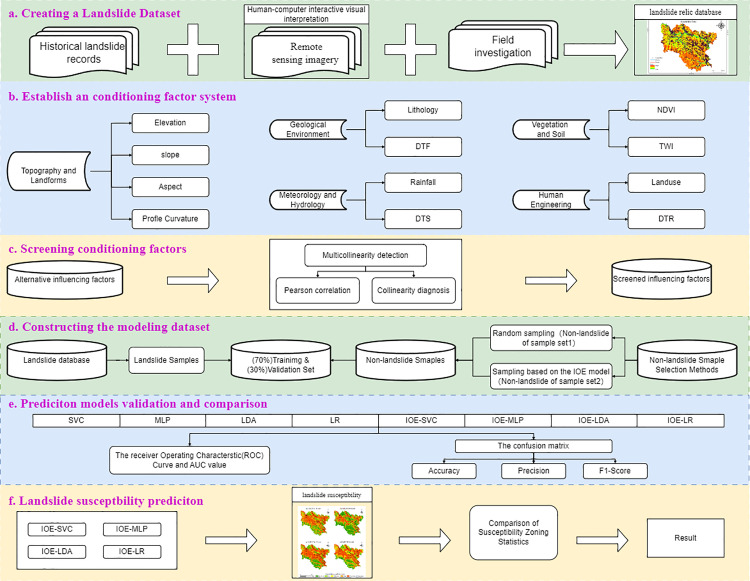
Flow chart of technical research.

The methodological flowchart for this study is presented in Fig 4.

#### (2) Assessment Procedure.

(a) Construction of a landslide inventory dataset. Landslide remnants were identified through human–computer interactive visual interpretation of Google Earth satellite imagery and then integrated with historical landslide records and field survey data to establish a comprehensive landslide inventory dataset.
(b) Identification of landslide conditioning factors: Twelve factors related to geological environment, geomorphology, meteorology and hydrology, vegetation and soil, and human engineering activities were selected to construct a landslide influencing factor system.
(c) Assessing the correlation of landslide conditioning factors: The potential multicollinearity among landslide conditioning factors was evaluated using the variance inflation factor (VIF) and tolerance, followed by Pearson correlation analysis to assess the strength of the correlation between each pair of variables.
(d) Constructing the modeling dataset: Datasets 1 and 2 were constructed according to the coupling method. Subsequently, each dataset was randomly split into training (70%) and testing (30%) subsets using the Python 3.11 programming language.
(e) Model development and evaluation: The attribute values of landslide conditioning factors were extracted into datasets 1 and 2. Using the Python 3.11 programming language, the SVC, MLP, LDA, and LR models were employed to train and test the data in datasets 1 and 2 at a 7:3 ratio. Model performance was assessed using AUC, accuracy (Acc), precision, and F1 score.
(f) Landslide susceptibility mapping and evaluation: The vector data generated in the previous step were converted to raster data using ArcGIS 10.8 software and classified into five categories using the natural breaks classification method. The classification performance and effect of each model and landslide susceptibility map were assessed using ROC curves and landslide frequency ratios to determine the most suitable model and optimal landslide susceptibility map for the study area.

## Results

### Multicollinearity diagnostics and pearson correlation analysis

**Fig 5:**
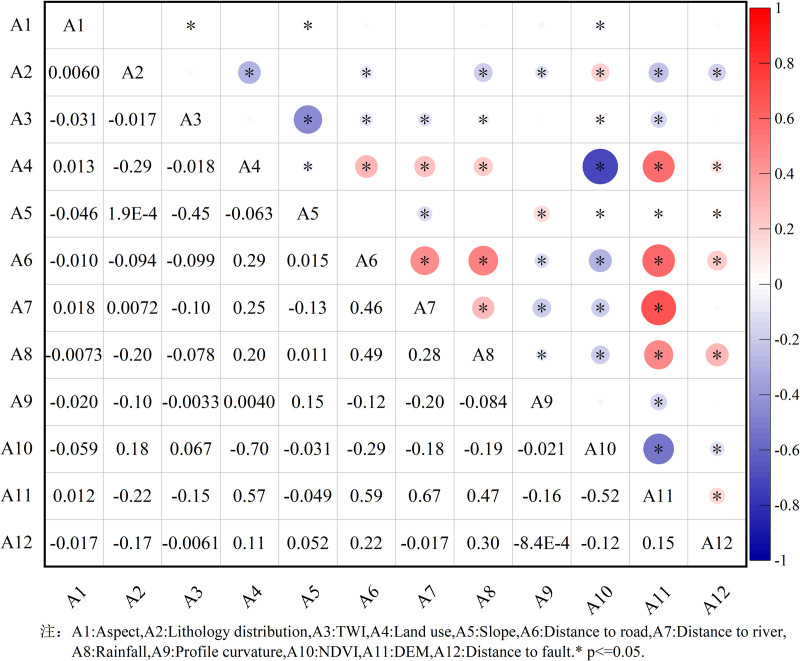
Correlation coefficient matrix.

Utilize ArcGIS 10.8 software to extract the attribute values of the 12 factors into the point attributes of the landslide dataset and export the attribute table. Perform collinearity diagnosis analysis among the factors using the linear regression analysis module in SPSS 27.0 software, as presented in Table 2. Conduct Pearson correlation analysis between each pair of factors using the correlation analysis module in SPSS 27.0, as illustrated in Fig 5.

**Table 2.** VIF and TOL of all conditioning factors.

Factors	Tolerances	VIF	Factors	Tolerances	VIF
Elevation	0.277	3.612	Rainfall	0.657	1.521
Slope	0.722	1.384	Distance to rivers	0.469	2.131
Aspect	0.987	1.013	NDVI	0.464	2.153
Profile curvature	0.904	1.106	TWI	0.750	1.334
Lithology	0.837	1.195	Land use	0.418	2.391
Distance to faults	0.846	1.182	Distance to roads	0.567	1.763

In the multicollinearity analysis of the data, the tolerance values for all 12 conditioning factors exceed 0.2, and the VIF values are below 5, suggesting that there is no multicollinearity among the factors.

In the Pearson correlation analysis, the Pearson correlation coefficients between all factors are below 0.8, suggesting that there is no strong correlation between any pair of conditioning factors.

### The relationship between conditioning factors and landslide relics

Utilize the “Extract Multi Values to Points” functionality in ArcGIS 10.8 to extract the attribute values of each conditioning factor to the landslide relic data points. Subsequently, tally the number of disaster points and grid cells for each level of the conditioning factors. Finally, employ an Excel spreadsheet to compute the values of a, b, FR_ij_, P_ij_, H_i_, H_i,max_, I_i_, P_i_, and W_i_ based on formulas (1) to (7) (Table 3).

**Table 3.** IOE model parametercal culation.

Factors	Classes	No. of landslides	NO.Raster	a	b	FR_ij_	P_ij_	H_i_	H_i,max_	I_i_	P_i_	W_i_
DEM	3153-3500	107	203879	0.0425	0.0227	1.8715	0.3156	0.5251	2.3219	0.0912	1.186	0.1082
	3500-4000	563	1126865	0.2238	0.1256	1.7816	0.3004	0.5212				
	4000-4500	933	2916445	0.3708	0.3251	1.1408	0.1924	0.4575				
	4500-5000	808	3301978	0.3211	0.3681	0.8726	0.1472	0.4068				
	>5000	105	1422193	0.0417	0.1585	0.2634	0.0444	0.1995				
Slope	0-15	395	2134018	0.157	0.2379	0.6601	0.1829	0.4483	2.3219	0.1536	0.7218	0.1109
	15-30	1476	4428231	0.5866	0.4936	1.1886	0.3293	0.5277				
	30-45	600	2195962	0.2385	0.2448	0.9744	0.27	0.51				
	45-60	45	204160	0.0179	0.0228	0.786	0.2178	0.4789				
	60-79	0	8989	0	0.001	0.0001	0	0.0004				
Aspect	Flat	4	24349	0.0016	0.0027	0.5859	0.0682	0.2643	3.1699	0.0263	0.9542	0.0251
	North	240	1308011	0.0954	0.1458	0.6544	0.0762	0.283				
	Northeast	316	1249772	0.1256	0.1393	0.9017	0.105	0.3414				
	East	232	1005584	0.0922	0.1121	0.8228	0.0958	0.3242				
	Southeast	365	1049069	0.1451	0.1169	1.2407	0.1445	0.4033				
	South	500	1198900	0.1987	0.1336	1.4872	0.1732	0.4381				
	Southwest	440	1145541	0.1749	0.1277	1.3697	0.1595	0.4224				
	West	258	940678	0.1025	0.1049	0.9781	0.1139	0.357				
	Northwest	161	1049456	0.064	0.117	0.5471	0.0637	0.2531				
Profile Curvature	0-0.5	584	2145494	0.2321	0.2391	0.9707	0.1872	0.4525	2.3219	0.0018	1.0372	0.0019
	0.5-1	860	3209089	0.3418	0.3577	0.9557	0.1843	0.4497				
	1-1.5	570	2008226	0.2266	0.2238	1.0122	0.1952	0.4601				
	1.5-2	291	968956	0.1157	0.108	1.071	0.2065	0.47				
	2-4.3	211	639595	0.0839	0.0713	1.1764	0.2268	0.4855				
Lithology	Hard Rock	164	1554255	0.0652	0.1732	0.3763	0.0878	0.3082	2.3219	0.0487	0.857	0.0417
	Loose Rock	5	31239	0.002	0.0035	0.5708	0.1332	0.3874				
	Harder Rock	500	1673218	0.1987	0.1865	1.0656	0.2487	0.4993				
	Weaker Rock	1195	3528036	0.475	0.3933	1.2079	0.2819	0.515				
	Weak Rock	652	2184612	0.2591	0.2435	1.0643	0.2484	0.4991				
Distance to faults	0-1000	730	2164114	0.2901	0.2412	1.2029	0.2453	0.4973	2.3219	0.0072	0.9808	0.0071
	1000-2000	558	1792466	0.2218	0.1998	1.1101	0.2264	0.4852				
	2000-3000	354	1393814	0.1407	0.1554	0.9057	0.1847	0.4501				
	3000-4000	228	1015109	0.0906	0.1131	0.801	0.1633	0.427				
	>4000	646	2605857	0.2568	0.2905	0.8841	0.1803	0.4456				
Distance to rivers	0-1000	334	782554	0.1328	0.0872	1.522	0.2701	0.5101	2.3219	0.011	1.1268	0.0124
	1000-2000	236	729452	0.0938	0.0813	1.1537	0.2048	0.4685				
	2000-3000	215	701359	0.0855	0.0782	1.0932	0.194	0.459				
	3000-4000	182	678651	0.0723	0.0756	0.9564	0.1698	0.4343				
	>4000	1549	6079344	0.6157	0.6776	0.9086	0.1613	0.4245				
Rain fall	574.9-598.5	684	1850462	0.2719	0.2063	1.3181	0.289	0.5176	2.3219	0.0351	0.9122	0.032
	598.5-610.5	912	2863946	0.3625	0.3192	1.1356	0.249	0.4994				
	610.5-624.8	557	2391226	0.2214	0.2665	0.8307	0.1821	0.4475				
	624.8-644.2	280	1205430	0.1113	0.1344	0.8284	0.1816	0.447				
	644.2-688.4	83	660296	0.033	0.0736	0.4483	0.0983	0.329				
NDVI	0.01-0.18	244	1884824	0.097	0.2101	0.4617	0.0918	0.3163	2.3219	0.0273	1.0056	0.0275
	0.18-0.33	384	1245576	0.1526	0.1388	1.0994	0.2187	0.4796				
	0.33-0.45	685	2110228	0.2723	0.2352	1.1576	0.2302	0.4878				
	0.45-0.57	754	2365126	0.2997	0.2636	1.1368	0.2261	0.485				
	0.57-0.92	449	1365606	0.1785	0.1522	1.1725	0.2332	0.4898				
TWI	5.10-8.70	725	3382073	0.2882	0.377	0.7645	0.1856	0.451	2.3219	0.0599	0.8236	0.0493
	8.70-10.59	1265	3856751	0.5028	0.4299	1.1696	0.284	0.5158				
	10.59-13.75	466	1282035	0.1852	0.1429	1.2962	0.3147	0.5249				
	13.75-18.16	52	378884	0.0207	0.0422	0.4895	0.1189	0.3652				
	18.16-28.17	8	71617	0.0032	0.008	0.3984	0.0967	0.326				
Land use	Forest	254	1644262	0.101	0.1833	0.5509	0.1114	0.3527	3.1699	0.2449	0.5496	0.1346
	Shrub	45	161355	0.0179	0.018	0.9945	0.201	0.4653				
	Grass	1994	5099231	0.7925	0.5684	1.3944	0.2819	0.515				
	Wetland	0	6036	0	0.0007	0.0001	0	0.0003				
	Cropland	1	2974	0.0004	0.0003	1.1991	0.2424	0.4956				
	Buildup	0	369	0	0	0.0001	0	0.0003				
	Snow/lce	68	1113569	0.027	0.1241	0.2178	0.044	0.1984				
	Bareground	154	931430	0.0612	0.1038	0.5896	0.1192	0.3658				
	Waterbody	0	12134	0	0.0014	0.0001	0	0.0003				
Distance to roads	0-1000	875	1946415	0.3478	0.217	1.6031	0.31	0.5238	2.3219	0.0279	1.0342	0.0289
	1000-2000	456	1584458	0.1812	0.1766	1.0263	0.1985	0.463				
	2000-3000	366	1351537	0.1455	0.1507	0.9657	0.1867	0.4521				
	3000-4000	291	1095728	0.1157	0.1221	0.9471	0.1831	0.4485				
	>4000	528	2993222	0.2099	0.3336	0.6291	0.1217	0.3697				

The weight value (W_i_) of the entropy index reflects the relationship between each conditioning factor and landslide relic data. A larger weight value signifies a more substantial contribution of the factor to landslides, and conversely, a smaller weight value indicates a lesser contribution. The calculation results demonstrate that in the entropy index model, the conditioning factors contributing to landslides in descending order are land use > slope > DEM > TWI > lithology > rainfall > distance to road > NDVI > aspect > distance to river > distance to fault > profile curvature. Land use, slope, and elevation significantly influence landslides, resulting in lower entropy values and higher weight values; in contrast, profile curvature and distance to fault have a minimal impact, leading to higher entropy values and lower weight values.

In terms of land use, the density of landslide debris is greatest in grasslands and farmlands, followed by shrublands and bare land. In high-altitude regions characterized by very high elevations, the proportion of farmland area is minimal, thus necessitating a focus on landslides in grasslands, shrublands, and bare land. Regarding slopes, areas with slopes of 15–30° exhibit the highest density of landslides and are most susceptible to landslides, whereas areas with slopes exceeding 60° experience virtually no landslides. In terms of elevation, within the study area, lower elevations are associated with higher landslide densities, while higher elevations are linked to lower densities.

The highest density of landslide debris in the study area is found between 3153 and 4000 m, largely due to the concentration of human activities in areas below 4000 m in elevation, which also align with regions where rivers and roads are densely located. Concerning the three distance parameters (distance to road, distance to fault, and distance to river), they demonstrate an inverse correlation with landslide debris, indicating that the closer the distance, the greater the propensity for landslides, with the highest incidence occurring within a 1km radius. The density of landslide debris is higher on slopes facing southeast, south, and southwest, as the study area is situated in the Northern Hemisphere, where south-facing slopes are sunny and more susceptible to landslides. Regarding rock strength, areas with weak rock, moderately weak rock, and moderately hard rock are more prone to landslides compared to areas with hard rock.

### Model evaluation

#### Accuracy analysis of single and coupled models.

**Fig 6:**
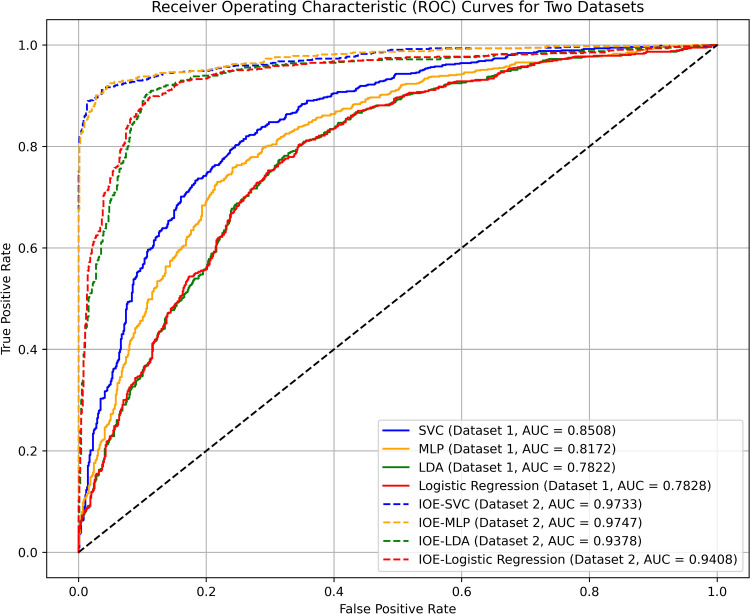
ROC curves of different models.

We employed the scikit-learn library in the Python 3.11 programming language to construct machine learning models. The datasets were divided into training and testing sets in a 7:3 ratio, and the SVC, MLP, LDA, and LR models were trained and tested on each dataset respectively. Precision of the models was validated by plotting the ROC curves, as shown in Fig 6. During the actual model construction process, using the default parameters may lead to some models failing to converge. Following extensive parameter tuning, the primary settings for each model were established as follows: For the SVC model, probability = True and random state = 42; for the MLP model, random state = 42, max iter = 1000, early stopping = True, and validation fraction = 0.1; for the LR model, max iter = 1000 and random state = 42; the LDA model used the default values. To avoid bias caused by imbalanced data distribution and to enhance the precision of model training, we employed the StratifiedKFold function for 5-fold cross-validation with the following parameters: n splits = 5, shuffle = True, and random state = 42. During the model training process, we calculated the 95% confidence intervals of AUC, Accuracy, Precision, and F1 values to evaluate the stability and reliability of the models, as presented in Table 4.

**Table 4.** The confidence intervals of AUC, Accuracy, Precision and F1 values for different models.

Model	AUC		Accuracy		Precision		F1	
	Value	CI.	Value	CI.	Value	CI.	Value	CI.
IOE-SVC	0.9733	[0.9708, 0.9804]	0.9385	[0.9200, 0.9488]	0.9836	[0.9464, 0.9823]	0.9343	[0.9174, 0.9470]
IOE-MLP	0.9747	[0.9567, 0.9735]	0.9365	[0.9126, 0.9316]	0.9655	[0.9283, 0.9517]	0.9332	[0.9106, 0.9299]
IOE-LDA	0.9378	[0.9274, 0.9442]	0.8882	[0.8730, 0.8950]	0.8942	[0.8675, 0.8929]	0.8851	[0.8724, 0.8955]
IOE-LR	0.9408	[0.9350, 0.9475]	0.8848	[0.8751, 0.9014]	0.9024	[0.8811, 0.8988]	0.88	[0.8726, 0.9020]
SVC	0.8508	[0.8539, 0.8838]	0.777	[0.7771, 0.8113]	0.7682	[0.7676, 0.8080]	0.7807	[0.7812, 0.8142]
MLP	0.8172	[0.8136, 0.8728]	0.7545	[0.7421, 0.8004]	0.7427	[0.7341, 0.7907]	0.7605	[0.7449, 0.8038]
LDA	0.7822	[0.7774, 0.7996]	0.722	[0.7076, 0.7321]	0.7176	[0.6992, 0.7245]	0.7251	[0.7073, 0.7396]
LR	0.7828	[0.7776, 0.8001]	0.722	[0.7048, 0.7340]	0.7182	[0.6956, 0.7250]	0.7248	[0.7058, 0.7432]

As depicted in Fig 6, the AUC, Accuracy, Precision, and F1 values of the IOE and machine learning coupled models are significantly higher than those of the individual models. The AUC values increased from a range of 0.7822 to 0.8508 to a range of 0.9378 to 0.9747, the precision increased from a range of 0.7176–0.7682 to a range of 0.8942–0.9836, accuracy increased from a range of 0.722–0.777 to a range of 0.8848–0.9385, and F1 scores increased from a range of 0.7248–0.7807 to a range of 0.88–0.9343. Among the individual models, SVC and MLP exhibited superior performance, with the SVC model performing the best. Among the coupled models, all four demonstrated excellent performance. The performance ranking from best to worst is IOE-MLP, IOE-SVC, IOE-LR, and IOE-LDA. Notably, the IOE-MLP and IOE-SVC models significantly outperformed the IOE-LR and IOE-LDA models.

As shown in Table 4, the relatively narrow confidence intervals of all models indicate good stability and reliability. Compared to individual models, the coupled models exhibit narrower confidence intervals, indicating superior stability and reliability.

#### Comparative analysis of model performance.

**Fig 7:**
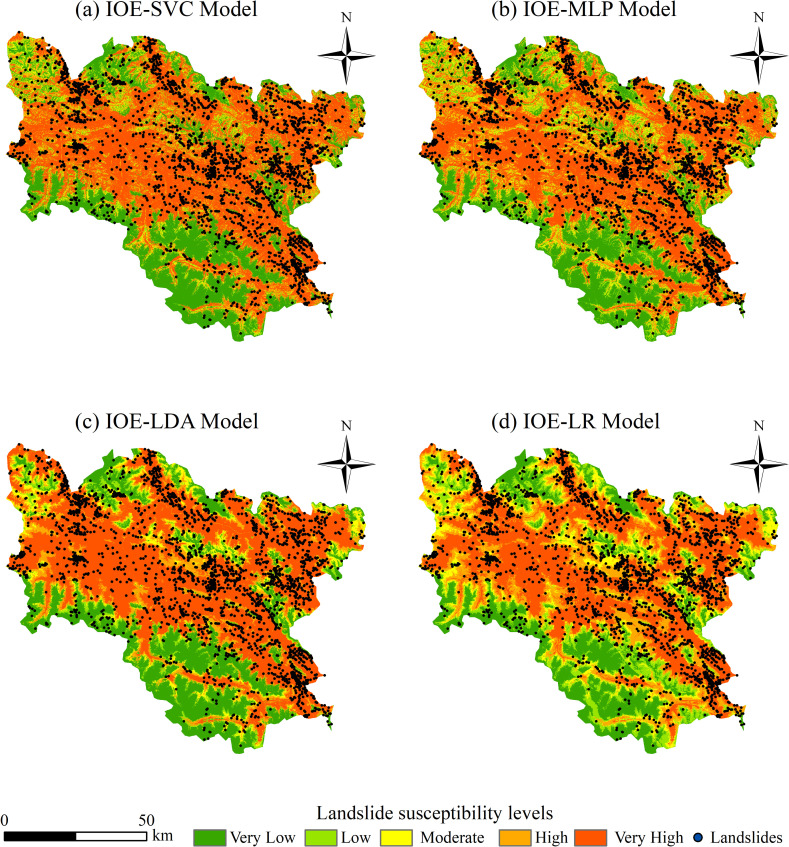
Vulnerability level diagram of different coupling models.

Given that individual models perform worse than coupled models, only the performance of coupled models is compared and analyzed in this section. Using the Python 3.11 programming language and the SVC, MLP, LDA, and LR models with dataset 2, we conducted a landslide susceptibility analysis for Luolong County and generated a landslide susceptibility map. Subsequently, the susceptibility map was classified into five levels—extremely high, high, moderate, low, and very low susceptibility—using the natural breaks classification method in ArcGIS 10.8, as shown in Fig 7. The original landslide points were overlaid on the susceptibility map to statistically analyze the number of landslide points and grid cells at each level, and to calculate the landslide density and landslide frequency ratio (the proportion of landslide points at each level divided by the proportion of the area at each level), as presented in Table 5.

**Table 5.** Classification performance of different coupling models.

Model	Landslide Susceptibility Zone Levels	Number of Grid Cells	Area Proportion	Number of Landslides	Landslide number ratio	Landslide Frequency Ratio
IOE-LDA	Very low	1968682	0.22	179	0.07	0.32
	low	455727	0.05	81	0.03	0.63
	middle	634570	0.07	154	0.06	0.87
	high	1238941	0.14	372	0.15	1.07
	Very high	4673440	0.52	1731	0.69	1.32
IOE-LR	Very low	1528129	0.17	117	0.05	0.27
	low	994456	0.11	149	0.06	0.53
	middle	1019448	0.11	264	0.10	0.92
	high	1937187	0.22	584	0.23	1.07
	Very high	3492140	0.39	1403	0.56	1.43
IOE-SVC	Very low	2086945	0.23	150	0.06	0.26
	low	517791	0.06	64	0.03	0.44
	middle	558101	0.06	50	0.02	0.32
	high	930956	0.10	101	0.04	0.39
	Very high	4877567	0.54	2152	0.85	1.57
IOE-MLP	Very low	1813910	0.20	93	0.04	0.18
	low	710494	0.08	99	0.04	0.50
	middle	764341	0.09	100	0.04	0.47
	high	1248072	0.14	236	0.09	0.67
	Very high	4434543	0.49	1989	0.79	1.60

As indicated by the results in Table 4, the landslide frequency ratios of the four coupled models decrease progressively from the extremely high susceptibility zones to the very low susceptibility zones. This suggests that the classification of landslide susceptibility by each coupled model is rational. Specifically, in the extremely high susceptibility zones, the ranking of landslide frequency ratios is as follows: IOE-MLP > IOE-SVC > IOE-LR > IOE-LDA. This indicates that the classification performance of the four models, from best to worst, is IOE-MLP, IOE-SVC, IOE-LR, and IOE-LDA. Overall, the coupled models outperform the individual models. The IOE-MLP and IOE-SVC models perform better than the IOE-LR and IOE-LDA models. The IOE-MLP model outperforms the IOE-SVC model.

Therefore, considering both model performance and classification effectiveness, the IOE-MLP model is the best.

### Spatial distribution of landslide susceptibility

As depicted in Fig 7, the spatial distribution of the evaluation results for the four coupled models (IOE-MLP, IOE-LR, IOE-SVC, and IOE-LDA) exhibit significant similarities. The very high susceptibility zones for landslides in Luolong County are predominantly aligned with river and road networks, particularly extending from the northwest to the southeast of the county, with additional concentrations observed in the western and southern regions. Several factors contribute to this spatial pattern: Firstly, the Nu River, which is the second largest river in Tibet, flows through the county from northwest to southeast. Additionally, numerous rivers such as Zhuomalongcuoqu and Daqu course through the western part, while Tongcuoqu, Dongcuoqu, Kangyucuo, and Baqu traverse the southern part. These riverine areas possess a high topographic wetness index, which facilitates landslide development. Secondly, Luolong County features prominently incised canyon topography, where rivers have deeply incised the landscape. In these areas, the rocks are soft, the elevation is low, and there is a prevalence of faults and well-developed fractures, all of which facilitate the formation of landslides. Thirdly, the average elevation of Luolong County is approximately 3859 m, which is quite high. Human settlements are typically located in the relatively lower-elevation river valleys, and most roads within the county are constructed along these valley areas. The frequent human engineering activities in these regions contribute to the occurrence of landslides. Fourthly, the southwestern part of Luolong County is dominated by the high-altitude Nyenchen Tanglha Mountains, while the northern part is characterized by the Tengri nor Mountain. These areas are primarily composed of high-altitude glaciers, where landslides are relatively rare. In contrast, the central region, which is predominantly grassland, is a zone where landslides are highly developed.

## Conclusion and discussion

### Discussion

#### Characteristics of landslide conditioning factors in Luolong county.

This study utilized imagery from the Google Earth platform, employing a human-computer interactive visual interpretation method to identify historical landslides in Luolong County, revealing a total of 2517 landslide scars of varying sizes. Landslides in this region are primarily influenced by geological settings, geomorphological background, and human engineering activities, representing the combined effect of multiple factors. The relative importance of the 12 factors investigated in this study is ranked as follows: land use type > slope > DEM > TWI > lithology > rainfall > distance to roads > NDVI > aspect > distance to rivers > distance to faults > profile curvature. Among these, land use type, slope, and elevation are identified as the dominant factors. Firstly, land use is one of the main causes of the high incidence of landslides in Luolong County. Due to the high altitude and cold climate of Luolong County, large trees with extensive root systems cannot thrive, and only a limited variety of grasses with shallow roots can adapt to the local environment. This results in sparse vegetation and a relatively fragile ecosystem[52]. Consequently, the vegetation types in Luolong County are distributed as follows: grasslands account for 79.25%, forests for 10.10%, glaciers for 2.70%, bare land for 6.12%, and other types for 1.83% of the total area. Secondly, slope is also one of the significant factors contributing to the susceptibility of landslides in Luolong County. The elevation in Luolong County ranges from 5476 m to 3153 m, with a difference of over 2000 m, and areas above 4000 m account for 85% of the total area. The greater the slope gradient and elevation difference, the more efficiently the potential energy of a landslide mass is converted into kinetic energy during movement, thereby increasing the velocity of the landslide[53]. Within the study area, regions with slopes between 15° and 30° comprise 58.66% of the area, while those between 30° and 45° account for 23.85%. This range of slopes is considered the most sensitive for landslides. Consequently, the significant elevation differences and the large proportion of areas prone to landslides are key reasons for the frequent occurrence of landslides, including high-velocity and long-runout landslides, in Luolong County. Thirdly, elevation is one of the primary factors promoting the development of landslides in Luolong County. The impact of elevation on landslides is not only evident in its influence on the stability of slopes through topography and geomorphology [54], but it also reflects the distribution characteristics of factors such as land use, distance to rivers and roads, and topographic wetness index.

#### Characteristics of landslide spatial distribution in Luolong county.

Spatial analysis reveals that landslides in Luolong County are extensively and densely distributed. The areas of very high and high susceptibility are predominantly located in the central part of the county, characterized by relatively low elevations, proximity to roads and rivers, and high soil moisture content. The susceptibility and density of landslides decrease with increasing distance from faults, rivers, and roads. Slopes facing southwest, south, and southeast are more prone to landslides, which is consistent with the findings of previous researchers[55,56]. High susceptibility areas are characterized by elevations below 4000 m, steep slopes, dense faulting, and weak to moderately weak geological lithology. They experience moderate rainfall, have a high density of human engineering activities, and are predominantly covered by grasslands. Luolong County, characterized by high elevation and significant topographic relief, is traversed by the Nu River and its tributaries, resulting in the formation of typical incised valley landforms [57]. The weak rock layers in Luolong County cover 73.61% of the total area and exhibit low strength and high deformability. They primarily consist of sandstone, siltstone, shale, conglomerate, and slate, which are susceptible to longitudinal and lateral erosion by rivers. This results in deep surface incision, significant elevation changes, and a high topographic wetness index. These regions often experience more intense tectonic activity, with frequent faults and earthquakes, leading to rock fragmentation and facilitating the development of landslides. Additionally, it is commonly accepted that elevations above 4000 m are unsuitable for long-term human habitation. With an average elevation of 3859 m in Luolong County, people typically reside along rivers, constructing their homes, roads, and living facilities on relatively lower-elevation terraces and gentle valley areas. Due to the inherent advantages of rivers with significant elevation drops for hydroelectric power generation, hydropower stations are also constructed in valley areas. Human engineering activities increase the susceptibility of these areas to landslides, resulting in high landslide susceptibility zones being located close to and along rivers. Additionally, these areas are also near roads and have relatively lower elevations. The very low susceptibility areas are primarily located in the Tengnate Mountain Range in the southwestern part of Luolong County, the Nyenchen Tanglha Mountain Range in the northern part, and some areas with relatively low slopes in the central region. These areas are characterized by elevations above 4500 m, perennial snow and ice cover, minimal human presence, and predominantly hard and moderately hard rock types, which are not conducive to the development of landslides.

#### Comparison with other studies.

Situated in a high-altitude and cold region, the southeastern Tibetan Plateau is characterized by high elevation, thin air, and sparse population, rendering geological surveys extremely challenging and the acquisition of samples highly difficult. Many researchers have focused on using algorithms and mapping units to enhance the accuracy of landslide susceptibility assessments. For instance, Mao Yimin et al. integrated SVM, RF, DT (Decision Tree), LR, FL (Fuzzy Logic), and TOPSIS to improve landslide susceptibility assessments in the Urmia Lake basin [13]. Yu Lanbing et al. applied LR, RF, SVC, and DL models to evaluate landslide-prone areas in the mid-to-upper reaches of the Three Gorges Reservoir region in Chongqing, China, achieving satisfactory results [45]. However, these studies often overlook the completeness and quality of samples. Incomplete samples can lead to insufficient information for training machine learning models, while poor-quality samples may cause misclassification, thereby affecting the final prediction outcomes. This study established a relatively comprehensive landslide database for Luolong County using remote sensing interpretation techniques and coupled the IOE model with machine learning models, optimizing the non-landslide samples during the coupling process. On the one hand, this approach enhanced the completeness of landslide samples and the quality of non-landslide samples, thereby improving prediction accuracy. On the other hand, the statistical learning model retained the spatial distribution characteristics of landslides, while the coupled model enhanced both prediction accuracy and precision.

Located in the Mediterranean-Himalayan seismic belt, the landslide influencing factors in Luolong County differ from those in inland China. Inland China is predominantly characterized by rainfall-induced landslides, with rainfall, elevation, and slope being the dominant factors. Areas with high susceptibility are typically found in mountainous and hilly regions with high rainfall, elevation, and slope [17,58,59]. In Luolong County, landslides are induced by non-rainfall. Among the 12 factors investigated in this study, the contribution of rainfall ranks sixth. The northern and northwestern regions, which receive the highest rainfall, are categorized as low and very low susceptibility zones. Conversely, some eastern areas with low rainfall are classified as high susceptibility zones. Rainfall increases soil moisture content, which can destabilize rock masses and promote landslide occurrence. However, it also supports the growth of water-demanding arboreal vegetation. The extensive root systems of these trees can stabilize slopes and reduce landslide likelihood. As a result, areas with dense forests in the northern and northwestern regions exhibit lower landslide susceptibility. Landslides in Luolong County are more earthquake-induced. The southeastern Tibetan Plateau, located at the collision zone between the Eurasian and Indian Ocean plates, is one of the most tectonically active and geologically complex regions globally [60,61]. Seismic factors significantly influence landslides in the southeastern Tibetan Plateau [62]. Over 50% of large- to mega-scale landslides along the Tibetan Plateau margin are triggered by paleo earthquakes or historical earthquakes [63]. The combined effect of heavy rainfall and strong earthquakes further increases the likelihood of landslide occurrence [64]. Faults are the primary sources of seismic activity, and fault density often indirectly reflects the frequency of earthquakes. The eastern region, with low rainfall, dense faults, low elevation, and intensive human engineering activities, exhibits high landslide susceptibility.

### Future research directions

Machine learning is data-driven and relies on large-scale training samples to support model training. In machine learning, small-sample issues are typically addressed through oversampling, which can, however, strengthen the information of a single class and lead to model overfitting. Sample imbalance primarily manifests as discrepancies between sample quantities and training data [65]. This issue can be addressed through oversampling or under sampling, both of which can either enhance or diminish certain class information, potentially leading to loss of information and exacerbating sample imbalance if not handled properly. This study employs the IOE model to optimize the sampling of non-landslide samples; however, it does not address the issue of sample imbalance. In recent years, researchers have explored various strategies to address sample imbalance. For instance, Yang Yajie et al. employed a sampling method based on Frequency Ratio (FR) and SBAS-InSAR interpretation results to improve sample quality [66]. Wang Lixia et al. utilized a Faster R-CNN landslide target detection method with multi-source imbalanced samples to address sample imbalance [65]. Liu Mengmeng et al. optimized samples using a fuzzy C-means method, thereby enhancing the training accuracy and reliability of landslide susceptibility models [67]. Wu Hongyang et al. effectively resolved sample imbalance in their study area and improved landslide prediction accuracy by optimizing samples using a hybrid sampling method coupled with semi-supervised classification and deep neural network models [68]. Given the diverse types and characteristics of samples, optimization methods vary accordingly. Future research can explore additional approaches to optimize samples, address small-sample and imbalance issues, and thereby enhance the prediction accuracy of machine learning models.

Landslide occurrence is closely related to the mechanical properties of rock masses, such as strength, deformation characteristics, and fracture development. Traditional landslide susceptibility assessments predominantly rely on empirical or physically-based mechanical models, which often face challenges such as difficulties in parameter acquisition and computational complexity in complex geological conditions. Yang Liu utilized rainfall data, topographic parameters, and hydrological parameters to determine the slope safety factor under rainfall conditions, thereby selecting safer non-landslide samples. This approach, which couples the TRIGRS physical model with the Random Forest algorithm, offers a novel perspective for predicting the susceptibility of shallow rainfall-induced landslides [69]. Integrating physically-based mechanical models with machine learning models to develop landslide susceptibility assessment methods that balance mechanical mechanisms and interpretability represents a promising direction for future research.

High-altitude cold regions are characterized by extensive deep permafrost layers, with some areas covered by snow and ice. Repeated freeze-thaw cycles lead to the destruction of soil structure and the degradation of mechanical properties, thereby triggering landslides. In recent years, some researchers have focused on this process. For instance, Guo Yanchen employed a Random Forest model to investigate the impact of freeze-thaw cycles on landslides in the Nanqian area of Qinghai, China, achieving satisfactory results [70]. With the rise in global temperatures, the accelerated melting of snow and ice, the degradation of permafrost, and the expansion of seasonal frozen ground have made the study of the relationship between freeze-thaw cycles and landslides in high-altitude cold regions an urgent research direction.

## Conclusion

The southeastern Tibet region is not only a crucial passage for the Sichuan-Tibet and Yunnan-Tibet railways but also a significant area for major water conservancy projects in China. It is part of the “Asian Water Tower” and the Three Rivers Source region and serves as a natural museum of geological disasters in China. This study takes Luolong County in southeastern Tibet as the research area, employing an optimized sampling strategy for non-landslide samples. It integrates the IOE model from statistics with various machine learning models to conduct landslide susceptibility mapping. The precision and effectiveness of each model are comprehensively assessed using AUC, Acc, Precision, and F1 scores. The conclusions drawn are as follows:

(1) Optimizing non-landslide samples using the IOE model reduces the probabilistic misclassification issues during the selection of non-landslide samples in machine learning models. Consequently, the IOE-coupled machine learning models (IOE-SVC, IOE-MLP, IOE-LDA, and IOE-LR) not only preserve the spatial characteristics of landslide evaluation inherent in statistical methods but also enhance prediction accuracy. The AUC, Acc, Precision, and F1 values of the four coupled models optimized with non-landslide samples are all higher than those of the single models before optimization. Specifically, the AUC values increased from a range of 0.7822 to 0.8508 to a range of 0.9378 to 0.9747, the precision increased from a range of 0.71760.7682 to a range of 0.8942–0.9836, accuracy increased from a range of 0.722–0.777 to a range of 0.8848–0.9385, and F1 scores increased from a range of 0.72480.7807 to a range of 0.88–0.9343. Among the four coupled models, the IOE-MLP model demonstrated superior performance, achieving the best overall performance and effectiveness. It provided a rational classification of landslide susceptibility zones, achieved the highest AUC value, and exhibited the best classification performance. Moreover, it had the highest landslide frequency ratio in the extremely high susceptibility zone, achieving the best classification effect.
(2) In terms of conditioning factors, landslides in Luolong County are influenced by a combination of geological environment, topography and geomorphology, and human engineering activities. They result from the interaction of multiple factors, with land use, elevation, and slope being the primary controlling factors.
(3) At the spatial scale, the areas of very high susceptibility to landslides in Luolong County are distributed around the Nu River and its tributaries, extending from the northwest to the southeast. This distribution aligns with the orientation of the Nu River, faults, and the trends of hard and weak rocks. The areas of very low susceptibility are predominantly found in the high-altitude, snow-covered regions surrounding the county.

In summary, optimizing non-landslide samples using the IOE model and subsequently employing machine learning coupled models for landslide susceptibility assessment yields superior results. We should focus on the impact of key conditioning factors such as land use, elevation, and slope, particularly in high mountain gorge areas that are closer to rivers and roads and have dense human engineering activities. In these areas of very high landslide susceptibility, it is crucial to enhance community-based prevention and control efforts, invest in advanced prediction and early warning hardware, and improve the capacity to respond to landslide disasters.
